# 

**Published:** 1892-10

**Authors:** 


*Entered at the Post Office at Austin, Texas, as Second Class Mail Matter.*

VOL. VIII. OCTOBER, 1893. No. 4.

### DANIEL'S MEDICAL JOURNAL

Subscription, $2.00 a Year, in Advance.—Single Copies, Twenty-five Cents.

F. E. DANIEL, M. D., Editor and Publisher, AUSTIN, TEXAS.

